# Supplementary material for: Comparative multiomics analysis of cell physiological state after culture in a basket bioreactor
Source: Sci Rep. 2022 Nov 23;12:20161. doi: 10.1038/s41598-022-24687-4 (PMC9686226; doi:10.1038/s41598-022-24687-4)
Supplement: Supplementary file 1 — Supplementary Information 1. [file 41598_2022_24687_MOESM1_ESM.zip › raw data/Transcriptomic raw data/6.Enrichment/1.GO/TE1vsTC1/GSEA/TE1/NUCLEOPLASM/NUCLEOPLASM(GO_0005654).html]

Details for gene set NUCLEOPLASM(GO:0005654)[GSEA]


|  || Dataset | readcount.sample |
| Phenotype | sample.cls |
| Upregulated in class | TE1 |
| GeneSet | NUCLEOPLASM(GO:0005654) |
| Enrichment Score (ES) | 0.40780553 |
| Normalized Enrichment Score (NES) | 1.8043907 |
| Nominal p-value | 0.0 |
| FDR q-value | 0.2128028 |
| FWER p-Value | 0.494 |
Table: GSEA Results Summary

  

Fig 1: Enrichment plot: NUCLEOPLASM(GO:0005654)
  
Profile of the Running ES Score & Positions of GeneSet Members on the Rank Ordered List

  

| PROBE | DESCRIPTION   (from dataset) | GENE SYMBOL | GENE\_TITLE | RANK IN GENE LIST | RANK METRIC SCORE | RUNNING ES | CORE ENRICHMENT || 1 | 103236678 | LOC103236678 |  |  | 700 | 6.610 | 0.0603 | Yes |
| 2 | 103245731 | ELL3 |  |  | 1736 | 2.263 | 0.0453 | Yes |
| 3 | 103222610 | TAF4B |  |  | 2022 | 2.072 | 0.0613 | Yes |
| 4 | 103241469 | MED12L |  |  | 2190 | 1.990 | 0.0814 | Yes |
| 5 | 103233022 | NUDT21 |  |  | 2321 | 1.926 | 0.1022 | Yes |
| 6 | 103224919 | ELL2 |  |  | 2437 | 1.882 | 0.1232 | Yes |
| 7 | 103214372 | GTF2F2 |  |  | 3426 | 1.622 | 0.1014 | Yes |
| 8 | 103245539 | GTF2A2 |  |  | 3567 | 1.597 | 0.1173 | Yes |
| 9 | 103242997 | LOC103242997 |  |  | 3716 | 1.571 | 0.1324 | Yes |
| 10 | 103242258 | MED31 |  |  | 3746 | 1.567 | 0.1528 | Yes |
| 11 | 103230666 | TADA1 |  |  | 3875 | 1.544 | 0.1685 | Yes |
| 12 | 103225096 | MEAF6 |  |  | 4171 | 1.506 | 0.1762 | Yes |
| 13 | 103245531 | ICE2 |  |  | 4424 | 1.470 | 0.1853 | Yes |
| 14 | 103244690 | TAF7 |  |  | 4742 | 1.431 | 0.1909 | Yes |
| 15 | 103242815 | MED13 |  |  | 4822 | 1.420 | 0.2070 | Yes |
| 16 | 103239198 | MED13L |  |  | 4848 | 1.417 | 0.2256 | Yes |
| 17 | 103231818 | MED14 |  |  | 4996 | 1.401 | 0.2384 | Yes |
| 18 | 103221505 | MED20 |  |  | 5106 | 1.390 | 0.2528 | Yes |
| 19 | 103242814 | INTS2 |  |  | 5353 | 1.363 | 0.2607 | Yes |
| 20 | 103229271 | MED6 |  |  | 5428 | 1.357 | 0.2762 | Yes |
| 21 | 103243867 | MED1 |  |  | 6025 | 1.300 | 0.2675 | Yes |
| 22 | 103245110 | MAML1 |  |  | 6508 | 1.262 | 0.2634 | Yes |
| 23 | 103220306 | STON1 |  |  | 6604 | 1.252 | 0.2765 | Yes |
| 24 | 103214287 | SUPT20H |  |  | 6634 | 1.249 | 0.2925 | Yes |
| 25 | 103248243 | MED17 |  |  | 7047 | 1.217 | 0.2909 | Yes |
| 26 | 103237144 | LOC103237144 |  |  | 7078 | 1.215 | 0.3064 | Yes |
| 27 | 103224965 | MED8 |  |  | 7374 | 1.192 | 0.3097 | Yes |
| 28 | 103236289 | MAML3 |  |  | 7443 | 1.186 | 0.3231 | Yes |
| 29 | 103235327 | MED19 |  |  | 7454 | 1.185 | 0.3391 | Yes |
| 30 | 103214895 | POLR3F |  |  | 7489 | 1.182 | 0.3539 | Yes |
| 31 | 103245565 | POLR2M |  |  | 7621 | 1.172 | 0.3643 | Yes |
| 32 | 103225252 | TAF12 |  |  | 7699 | 1.166 | 0.3770 | Yes |
| 33 | 103225226 | LOC103225226 |  |  | 8014 | 1.142 | 0.3788 | Yes |
| 34 | 103215450 | POLR3D |  |  | 8526 | 1.116 | 0.3714 | Yes |
| 35 | 103214401 | MED4 |  |  | 8713 | 1.103 | 0.3784 | Yes |
| 36 | 103214967 | MED10 |  |  | 9599 | 1.048 | 0.3533 | Yes |
| 37 | 103229444 | GTF2A1 |  |  | 9653 | 1.044 | 0.3654 | Yes |
| 38 | 103223371 | EP300 |  |  | 9745 | 1.038 | 0.3757 | Yes |
| 39 | 103245617 | LEO1 |  |  | 9893 | 1.029 | 0.3834 | Yes |
| 40 | 103231770 | LOC103231770 |  |  | 10273 | 1.006 | 0.3803 | Yes |
| 41 | 103249020 | TAF4 |  |  | 10825 | 0.968 | 0.3691 | Yes |
| 42 | 103229907 | LIN52 |  |  | 11104 | 0.950 | 0.3698 | Yes |
| 43 | 103243722 | FAAP100 |  |  | 11580 | 0.921 | 0.3613 | Yes |
| 44 | 103244860 | MED7 |  |  | 11585 | 0.921 | 0.3739 | Yes |
| 45 | 103222970 | MED15 |  |  | 11789 | 0.907 | 0.3774 | Yes |
| 46 | 103234670 | PAF1 |  |  | 11860 | 0.902 | 0.3868 | Yes |
| 47 | 103239694 | MED22 |  |  | 11890 | 0.900 | 0.3979 | Yes |
| 48 | 103234668 | MED29 |  |  | 11948 | 0.897 | 0.4078 | Yes |
| 49 | 103232147 | MED12 |  |  | 12883 | 0.840 | 0.3777 | No |
| 50 | 103225264 | MED18 |  |  | 14582 | 0.734 | 0.3118 | No |
| 51 | 103242195 | MED11 |  |  | 16268 | 0.623 | 0.2451 | No |
| 52 | 103234183 | ELL |  |  | 16926 | 0.575 | 0.2236 | No |
| 53 | 103245615 | LOC103245615 |  |  | 17570 | 0.527 | 0.2022 | No |
| 54 | 103242473 | MED9 |  |  | 17702 | 0.515 | 0.2034 | No |
| 55 | 103223797 | LOC103223797 |  |  | 17964 | 0.492 | 0.1986 | No |
Table: GSEA details
[plain text format]

  

Fig 2: NUCLEOPLASM(GO:0005654)
  
Blue-Pink O' Gram in the Space of the Analyzed GeneSet

  

Fig 3: NUCLEOPLASM(GO:0005654): Random ES distribution
  
Gene set null distribution of ES for
 **NUCLEOPLASM(GO:0005654)**
